# Persistence of Correlations in Neurotransmitter Transport through the Synaptic Cleft

**DOI:** 10.3390/biology13070541

**Published:** 2024-07-18

**Authors:** Masroor Khonkhodzhaev, Shota Maglakelidze, Yonatan Dubi, Lev Mourokh

**Affiliations:** 1 Physics Department, Queens College, City University of New York, Flushing, NY 11367, USA; masroor.khonkhodzhaev14@qmail.cuny.edu; 2 Edward R Murrow High School, Brooklyn, NY 11230, USA; 3 Department of Chemistry and the Ilse-Kats Center for Nano-Science, Ben-Gurion University of the Negev, Beer-Sheva 8410501, Israel

**Keywords:** synapse, neurotransmitters, correlations, diffusion coefficient, quantum brain

## Abstract

**Simple Summary:**

The human brain is still an enigma, and the relation between its physiology and function is still poorly understood. Advancements in quantum computations led to the hypothesis that neurons can also exercise quantum coherence, and a model for neuronal activity with quantum correlations appearing from the nuclei entanglement was proposed. However, whether such correlations can survive in a high-temperature biological environment to propagate through a neural network is still an open question. In this work, we examine one of the first steps in this process, the diffusion of neurotransmitters through the synaptic cleft, and show that, at least at this step, the correlations are persistent. Our results do not confirm the “quantum brain” hypothesis, but at least, they do not disprove it either, with studies on further steps needed.

**Abstract:**

The “quantum brain” proposal can revolutionize our understanding of cognition if proven valid. The core of the most common “quantum brain” mechanism is the appearance of correlated neuron triggering induced by quantum correlations between ions. In this work, we examine the preservation of the correlations created in the pre-synaptic neurons through the transfer of neurotransmitters across the synaptic cleft, a critical ingredient for the validity of the “quantum brain” hypothesis. We simulated the transport of two neurotransmitters at two different clefts, with the only assumption that they start simultaneously, and determined the difference in their first passage times. We show that in physiological conditions, the correlations are persistent even if the parameters of the two neurons are different.

## 1. Introduction

### 1.1. Quantum Biology

Quantum mechanics (QM) are the set of laws describing the behavior of elementary particles—electrons, atoms, etc. As opposed to “classical mechanics” (e.g., Newton’s laws), in which particles are described by their position, velocity, and acceleration, and interact with each other through “forces” (related to acceleration through Newton’s second law), in QM (at least in the common interpretation of QM), particles are described by a “wave function”—an abstract quantity that describes the probability of a particle to have a specific position, velocity, etc. This probability function obeys a wave equation called “Schrodinger’s equation”, and its measurement leads to a “choice” or “manifestation” of a certain possibility, with probability related to the numerical value of the wave function at this possibility. As unbelievable as it sounds, QM have proven incredibly useful in describing various measurements of elementary particles and has led to explanations of numerous physical effects, from the structure of the atom through the properties of the periodic elements to magnetism and superconductivity and more.

Two central features of quantum mechanics, which come directly from describing particles through a wave function, are the superposition principle and the concept of entanglement. The superposition principle asserts that a particle can occupy a wave function that reflects very different characteristics simultaneously. A famous example is the “spin”, a kind of internal rotation that can be either clockwise or counterclockwise. A basketball (clearly a “classical” object) can only spin either in one direction or the other. However, an electron, being a quantum particle, can be in a state where both clockwise and counterclockwise are simultaneously possible, and only by measuring the spin will the electron “choose” one (with a probability encoded in the wave-function values). Entanglement is the property. The second feature, entanglement, is a phenomenon where two particles become linked through a “joint wave function” so that the state of one particle instantly influences the state of the other, no matter how far apart they are.

But quantum mechanics has its limitations, exemplified by Schrodinger himself in his famous “Schrodinger’s cat” thought experiment: If we put a cat in a closed box and put in the box a gas molecule that is in a quantum superposition of “toxic” and “benign” states, can we say that the cat is in a superposition of “dead” and “alive”? The answer, as Schrodinger pointed out himself, is “of course not”—cats are not quantum particles. Cats are “classical” and cannot be in a state of superposition because they are composed of many particles that interact in an “incoherent” way, thus breaking the coherent wave-like properties of the wave function. This is a hard point, so here is an analogy: Consider an ocean wave. As it rises, the many particles of water move together to form a smooth waveform, which moves coherently (i.e., it keeps its waveform for a long time). As the wave nears the shore, it starts to interact with its environment (e.g., the ocean floor) until the wave breaks and turns into foam—the coherent motion is broken into many waves that are moving incoherently, forming the mixed wave we see as the waves break.

This analogy (not perfect, as all analogies are, but at least helpful) can thus lead us to an essential characteristic of quantum systems called “coherence time”, which is defined as the time a particle can propagate in a coherent wave function (obeying the dynamics of Schrodinger’s equation) before the environment ruins the coherence and “mixes” the wave. The coherence time is the reason that quantum mechanics are typically observed in small, cold, and isolated systems because when a system is “hot and wet”—that is, when the particle is in contact with an environment that has many particles in it (like a liquid), it is wet, and it is also hot because the particles in the environment move quickly and in an uncorrelated way—there is little chance of observing quantum phenomena such as superposition and entanglement.

That is why, typically, when we describe biological systems, we do not think of quantum mechanics (a point already raised by Schrodinger himself in his book, *What is Life?* [1]). The systems are simply too hot and wet, and the coherence time is, thus, many orders of magnitude smaller than any relevant biological timescale [2].

Nonetheless, the idea that quantum effects might be significant in some biological systems has been discussed many times over the years [3]. In recent years, there has been renewed interest in the concept of quantum biology [4,5,6], which came mainly from the discovery of wave-like dynamics in the absorption patterns of certain photosynthetic systems [7], giving rise to the idea that some photosynthetic systems evolved to operate in a regime that is “intermediate”, i.e., between quantum and classical (or equivalently, where the coherence time is not infinite, but not too short), so their dynamics are partially quantum. Why? It turns out that in many cases, adding a small amount of incoherence to a quantum system may actually increase its efficiency (a situation dubbed “environment-assisted quantum transport”, or ENAQT) [8,9,10,11], and the claim was that photosynthetic systems take advantage of this effect to increase their yield.

Since then, many arguments have been made against the role of quantum effects in photosynthetic systems [12,13], but the idea of natural systems using quantum properties, namely superposition and entanglement, to achieve certain functionality or to enhance efficiency took hold. Various systems, from olfaction through protein transfer to the navigation system of migratory birds, were suggested to utilize quantum effects [5]. But probably, the most exciting, and surely the most imaginative and captivating idea, is that of the quantum brain.

### 1.2. Quantum Brain

**Figure 1:**
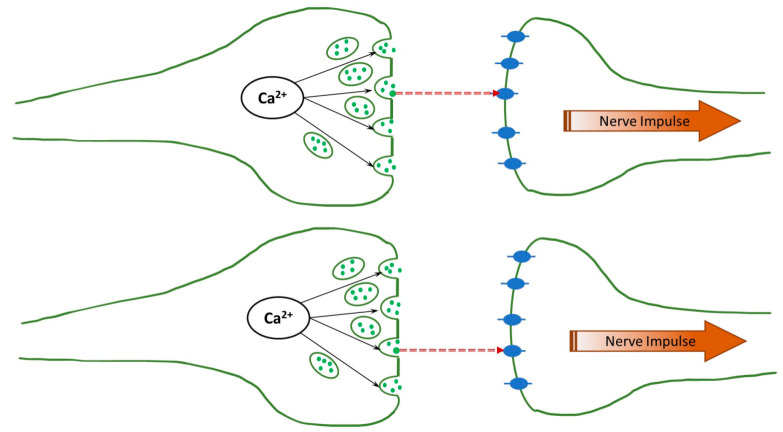
Schematics of the synapse. Calcium ions activate the neurotransmitters (shown as green solid circles), which are transferred across the synaptic cleft through one-dimensional diffusion and, in turn, activate the ligand-gated ion channels (shown as blue ovals).

The human brain is an immensely complex, still poorly understood device. While much has been learned about its physiology, relatively little is known about its operation principles, i.e., how the physiological function is translated into “computation”, such as thinking, memory, and even consciousness at a deeper level. The complexity is easy to understand: The brain involves more than 80 billion neurons that communicate using electrical and chemical signaling, and even the (electro-chemical) interaction between any two neurons is immensely complicated. A synapse is the point of communication between two neurons (pre-synaptic and post-synaptic). An electric signal (action potential) propagates along the pre-synaptic neuron, and at the end of the axon, near the synapse, it activates the voltage-gated ion channels, and the Ca^2+^ ions are injected into the neuron. These ions release the neurotransmitters (with glutamates being the most common), which diffuse across the synaptic cleft, as can be seen in Figure 1. After reaching the post-synaptic neuron, they activate the ligand-gated ion channels, leading to the firing (the formation of the action potential) of the neuron. 

Among the models relating physiology to function, the most successful is the Hopfield network (or the Ising model of a neural network) [14,15], in which neurons are represented as “classical bits” (or classical spins, namely elements with two degrees of freedom—off/on, idle/firing, etc.) that are interconnected with each other with different (and adjustable) weights. The “spins” represent the state of a neuron (“idle” or “firing”), and the weighted connections represent the connections between the neurons. The dynamics are determined by determining the state of each neuron according to the state of all its neighbors, weighted adequately by the connections between them, and the state of each neuron is updated during the dynamics until a steady state is reached. The neural function is determined by “learning”, whereby a set of pre-defined states (i.e., “images” or “memories”) is used to determine the weights of the network. Once the learning process is completed, a given initial state of the network will dynamically flow toward the closest initial input. In other words, the network will move from a “fuzzy image” toward the “remembered image”, thus mimicking associative memory operation. The Hopfield model has also been central in developing machine learning technology [16].

Recently, an additional layer of complexity has been added to the discussion about the brain’s function–operation connection in the form of suggestions that the brain may operate, at some level, using quantum effects. The possibility that such a network can have a quantum character changes perspectives of the brain entirely; it requires completely different approaches and should be carefully explored.

The idea that quantum correlations play a role in the brain’s functioning was first proposed by Penrose [17]. It was swiftly dismissed because of the common belief that such correlations cannot survive in the “hot and wet” environment [2]. Recently, the notion of a quantum brain was revived by Fisher, who suggested that quantum correlations can be stored in nuclear spins with an extremely long decoherence time (in contrast to microtubules by Penrose and Hameroff [18]). Fisher proposes the following [19]. When the adenosine triphosphate (ATP) molecule is dissociated into adenosine monophosphate (AMP) and pyrophosphate ion (P_2_O_7_)^4−^, the two phosphorous nuclei are in the singlet state. These nuclei remain entangled when the pyrophosphate ion is subsequently dissociated into two phosphate ions (PO_4_)^3−^. Then, they join two different Posner molecules Ca_9_(PO_4_)_6_. These Posner molecules appear in two distinctive pre-synaptic neurons and melt simultaneously because of the entanglement. This leads to the simultaneous injection of Ca^2+^ ions (instead of the voltage-gated ion channels of the standard model) and the activation of the neurotransmitters in the two pre-synaptic neurons, introducing inter-neuronal correlations, which are assumed to be transferred to the post-synaptic neurons and their correlated firings. In other words, Fisher suggests that quantum correlation between phosphorous nuclear spins may lead to a correlation in operation between different neurons. Later, Halpern and Crosson showed [20] that such correlations enable full-scale quantum computations in the brain. Although it was recently demonstrated [21] that the Posner molecule, as a trimer, is not stable enough to host a biological qubit, the corresponding dimer can serve instead [21].

According to Fisher’s proposal, correlations between the pre-synaptic neurons lead to correlations between the firings of the post-synaptic neurons. However, there is a significant gap between these two events. Activated neurotransmitters are transferred across the synaptic cleft to the post-synaptic neurons, where they activate the ligand-gated ion channels; see Figure 1. Associated charge accumulation leads to the formation of the action potential and, eventually, to the post-synaptic neuron firing. Correspondingly, the correlations must persist through the neurotransmitter transfer and the development of the action potential.

A natural question thus arises: Because (even within Fisher’s model) the transfer of the neurotransmitters across the synaptic cleft is a classical random-walk type process, when taking physiological parameters into account, does a quantum-correlated (or even just simultaneous) release of neurotransmitters lead to correlations between the corresponding post-synaptic neuron firing? Answering this question is crucial because if the answer turns out to be negative, then quantum correlations, even if they are present at the level of pre-synaptic neurons, will vanish as the neurotransmitters cross the synaptic cleft and will not lead to additional long-range correlations throughout the neuronal network.

In the present paper, we thus address this question and show that correlations indeed seem to be maintained as neurotransmitters cross the synaptic cleft. We simulate the transfer of two glutamate neurotransmitters through the synaptic clefts of the same widths, as shown in Figure 1, and determine their first passage times. The results are compared to the analytical predictions for the real-valued, continuous-time stochastic (Wiener) process. We demonstrate that the correlations are persistent when the diffusion coefficients have physiological values, even if they differ for the two clefts. This can be seen in the histogram of differences in the first passage times exhibiting a clear bunching form. However, when one or both diffusion coefficients are small, the histogram is smeared, and, in other words, the correlations are lost. The difference in the voltages does not affect the histogram unless it is unphysiologically large.

## 2. Methods

The dynamics of the neurotransmitters within the synaptic cleft in the overdamped regime obey the classical Langevin equation:

*ζ dx/dt* = *F* + *ξ*(*t*), (1)

where *ζ* is the viscosity, *F* is the external force, and *ξ*(*t*) is the fluctuation force with a zero mean value and the variance given by

〈*ξ*(*t*) *ξ*(*t*_1_)〉 = 2 *ζ T* δ(*t* − *t*_1_). (2)

For glutamate neurotransmitters with charge −*e*, the external force is

*F* = −*e V*⁄*d* + *F_W_*, (3)

where *V* is the (negative) voltage across the synaptic cleft, *d* is its width, and *F_W_* is the force preventing the neurotransmitter from returning to the pre-synaptic neuron. For our simulations, we used the cleft width *d* = 20 nm and the default values of the diffusion coefficient (related to the viscosity as $D=T/\zeta ,\;k_{B}=1$) and voltage as *D* = 0.33 μm^2^/ms [22] and *V* = −4 mV [23], respectively.

**Figure 2:**
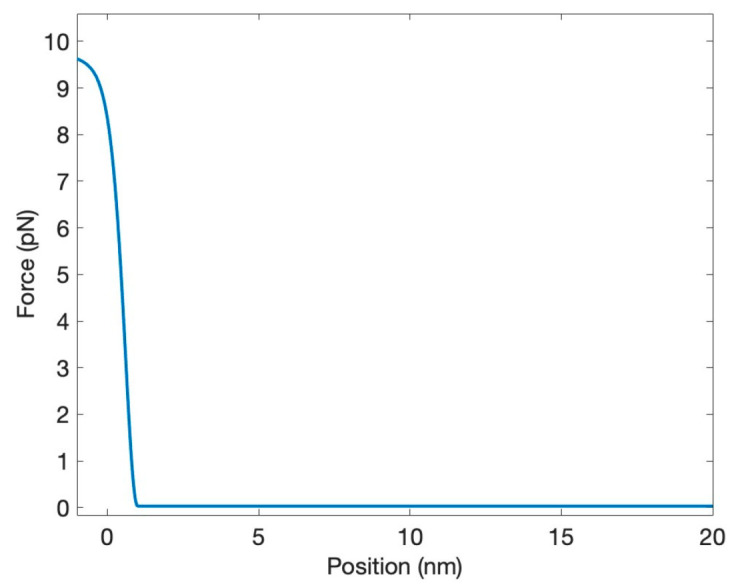
The soft-wall force preventing the neurotransmitter from returning to the pre-synaptic neuron.

We modeled the force FW as

(4) $$F_{W}=4F_{MAX}\left(\frac{exp\left(\frac{x-x_{0}/2}{l_{C}}\right)}{{\left(exp\left(\frac{x-x_{0}/2}{l_{C}}\right)+1\right)}^{2}}-\frac{exp\left(\frac{x+x_{0}/2}{l_{C}}\right)}{{\left(exp\left(\frac{x+x_{0}/2}{l_{C}}\right)+1\right)}^{2}}\right)-F_{MAX},$$

where *F_MAX_* is the maximal value of the force, *x*_0_ is the effective width of the force region, and *l_C_* is the steepness. This force should be “impenetrable” to prevent the return of neurotransmitters to the pre-synaptic cleft. At the same time, it must be “soft”, i.e., a strong reflection should not occur. In our model, it was achieved for the following set of parameters: *F_MAX_* = 9.6 × 10^−12^ N, *x*_0_ = 2 × 10^−9^ m, and *l_C_* = −3 × 10^−10^ m, with the resulting shape shown in Figure 2.

**Figure 3:**
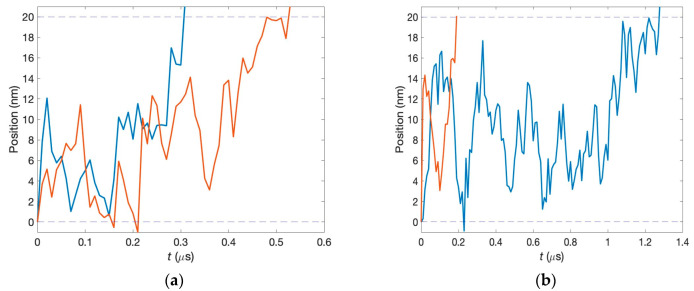
Characteristic time dependencies of the two neurotransmitter positions for different simulation runs. (**a**) The first passage times are close to each other. (**b**) The first passage times are very different.

The first passage times are determined from the numerical procedure modeled in MATLAB 9.13 (R2022b). In each run, the two neurotransmitters start simultaneously from the corresponding pre-synaptic neurons. Their dynamics inside the synaptic clefts are governed by Equation (1). Even if the two clefts have the same parameters, the motion of neurotransmitters is disparate because of fluctuations. We model this using different realizations of the fluctuation forces. The first passage times, $t_{1}$ and $t_{2}$, are recorded when each neurotransmitter reaches the post-synaptic neuron, and their difference, $∆T_{1P}=t_{1}-t_{2}$, is calculated. Two specific examples are shown in Figure 3. The upper panel exhibits the situation in which the first passage times are close, while for the lower panel, these times are very different. After many runs, the corresponding histogram is formed. For our calculations, 50,000 runs were used, and the time step of our simulations was 10 ns.

The outcomes of our simulations can be compared to analytical results. For the Wiener process in one dimension, the probability distribution $\gamma \left(t\right)$ for the first passage time can be expressed in terms of the parameters of Equations (1)–(3) as [24]

(5) $$\gamma \left(t\right)=\frac{d}{\sqrt{4\pi Dt^{3}}}\exp\left(\frac{{\left(d+eVt/\zeta d\right)}^{2}}{4Dt}\right).$$

This probability distribution allows us to calculate the variance of Δ*T*_1*P*_ as

(6) $$\left\langle \delta^{2}\right\rangle =\int \int {dt_{1}dt_{2}\left(t_{1}-t_{2}\right)}^{2}\;\gamma \left(t_{1}\right)\gamma \left(t_{2}\right)=4\;{\left(\frac{T}{e\left|V\right|}\right)}^{3}\;\frac{d^{4}}{D^{2}},$$

and the corresponding standard deviation has the form

(7) $$\delta =\sqrt{\left\langle \delta^{2}\right\rangle }=2\;{\left(\frac{T}{e\left|V\right|}\right)}^{3/2}\;\frac{d^{2}}{D}.$$

## 3. Results

**Figure 4:**
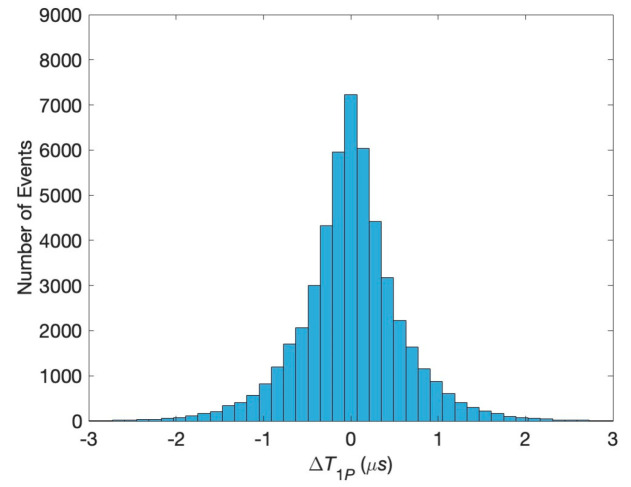
Histogram of the difference in the first passage times for neurotransmitters at physiological conditions.

The histogram for the difference in the first passage times at physiological conditions specified above is shown in Figure 4. It has a pronounced sharp maximum, indicating the high probability of the simultaneous reaching of the post-synaptic neuron. This means that the initial correlations of the pre-synaptic neurons persist through the transfer of neurotransmitters across the synaptic cleft. This is the main result of our study.

**Figure 5:**
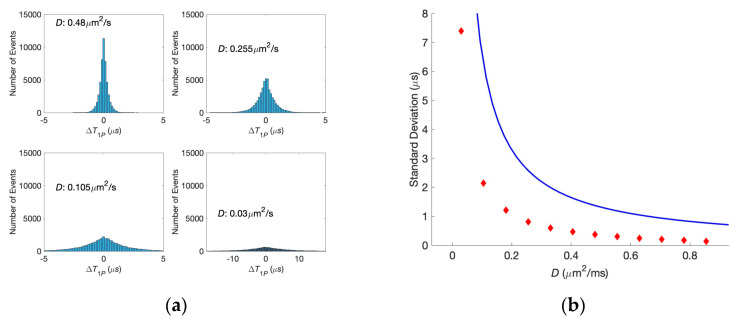
(**a**) Histogram of the difference in the first passage times at various diffusion coefficients (the same for both synapses). (**b**) Dependence of the standard deviation on the diffusion coefficient for the analytical solution (blue solid line) and the histograms (red symbols).

We performed the same analysis for various diffusion coefficients to find the conditions in which the correlations are lost. Corresponding histograms are shown in Figure 5a. It is evident from this figure that the decreasing of the diffusion coefficient leads to the smearing of the histogram, the synapses become less and less correlated, and, finally, at the diffusion coefficient *D* = 0.03 μm^2^/ms, the correlations are entirely lost, i.e., equal first passage times for the two neurotransmitters and their significant difference have almost the same probabilities. The dependence of the standard deviation of the histogram on the diffusion coefficient is shown in Figure 5b, where the analytical estimate is also present. One can see that the simulation results and theoretical predictions based on the Wiener process have the same shape, although they are slightly off quantitatively. These quantitative differences are caused by force *F_W_*, preventing the particle motion in the negative direction. At the same time, an analytic result is obtained for a symmetric random walk (where the particles can move in either positive or negative directions). It should be noted that introducing this force decreases the diffusion time.

**Figure 6:**
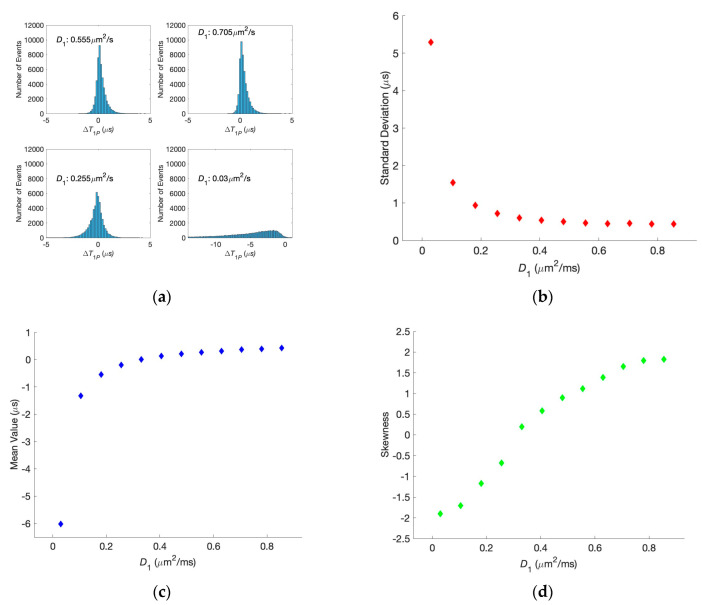
(**a**) Histogram of the difference in the first passage times for different diffusion coefficients with *D*_2_ = 0.33 μm^2^/ms. (**b**–**d**) Dependencies of the standard deviation, mean value, and skewness of the histograms, respectively, on one of the diffusion coefficients, when the second one is 0.33 μm^2^/ms.

In Figure 6a, we show the histograms for the situations in which the diffusion coefficient for one of the synaptic clefts is kept at physiological conditions while the second one is modified. One can see that the increase in just one of the diffusion coefficients does not lead to the histogram broadening. At the same time, its decrease below the physiological value affects the histogram width, similar to the situations in Figure 5. The dependencies of the standard deviation, mean value, and skewness of the histogram on the changing diffusion coefficient are shown in Figure 6b–d, respectively. For significant differences, the histograms are not symmetric anymore, as can be seen in the skewness. A similar analysis for different applied voltages shows that the broadening also does not happen, and the histogram asymmetry becomes visible only at unphysiologically large voltages.

## 4. Discussion

Using numerical simulations and analytical results, we have examined the correlation between the passage times of diffusing neurotransmitters with correlated activation, i.e., particles that start their diffusion simultaneously. For physiological parameters relevant to transport across the synaptic cleft, we show that correlated activation leads to the correlations in the first passage times, corresponding to the correlated initiation of the ligand-gated ion channels in the post-synaptic neurons. Put simply, we show that if neurotransmitters are simultaneously injected into the synaptic cleft of two separate neurons, the resulting post-synaptic neuronal firing can also be (almost) simultaneous. This is a non-trivial result, keeping in mind that the neurotransmitters drift–diffuse across the synaptic cleft and that the pathways of random-walking particles can very quickly become uncorrelated even if they start the walk simultaneously. Therefore, our results support, to some extent, Fisher’s hypothesis that quantum correlations between phosphor nuclei can lead to the simultaneous firing of different neurons. It should be noted, however, that our results are valid even if the quantum correlations in pre-synaptic neurons arise by arbitrary mechanisms, not necessarily the one proposed by Fisher.

However, some additional points must be addressed before we can proclaim that the “quantum brain” is feasible. First, our calculations were performed in one dimension, although the synaptic cleft is three-dimensional. However, in the case of the one-dimensional drift under the applied voltage, we do not expect a qualitative change in the results. For example, even if the powers appearing in Equation (7) are different in three dimensions, we still expect the standard deviation to be a monotonically decreasing function of the diffusion coefficient. Second, the correlations should persist in subsequent events before the firing of the post-synaptic neurons. This includes the activation of the ligand-gated ion channels and the formation of the action potentials. Finally, even if it is found that adjacent neurons fire in a correlated way due to initial quantum entanglement, it is still not clear what role such correlations would play in performing the computational tasks required for a functioning brain. We do not know much about the interplay between the physiological operation of neurons and their functional operation. Whether quantum correlations play a role remains to be validated, but our results show that it cannot be ruled out.

## 5. Conclusions

In conclusion, we considered two neurotransmitters simultaneously activated in two different pre-synaptic neurons to diffuse through the synaptic clefts. This can be related to the case in which the simultaneous activation is caused by quantum entanglement, i.e., we effectively examined the feasibility of the “quantum brain” hypothesis. We showed that under physiological conditions, these correlations survive the classical diffusion process, which is the first event in the multi-step dynamics of the neural network.

## Data Availability

The MATLAB codes used for this article will be made available by the authors without undue reservation.
